# Supplementary figures and images for: Long-term exposure to low concentrations of polycyclic aromatic hydrocarbons and alterations in platelet indices: A longitudinal study in China
Source: PLoS One. 2022 Nov 2;17(11):e0276944. doi: 10.1371/journal.pone.0276944 (PMC9629616; doi:10.1371/journal.pone.0276944)

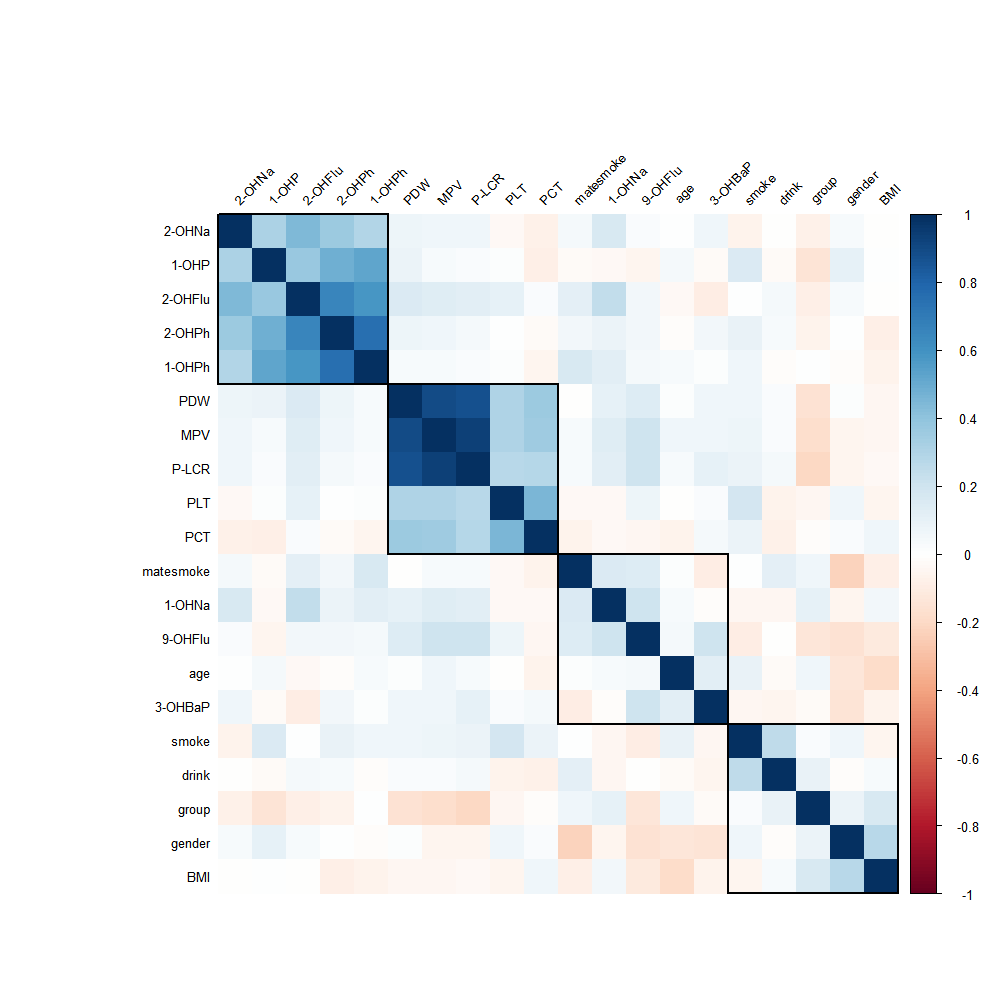

Supplement: S1 Fig — (TIF) [file pone.0276944.s001.tif]
