# Supplementary material for: Long-term exposure to low concentrations of polycyclic aromatic hydrocarbons and alterations in platelet indices: A longitudinal study in China
Source: PLoS One. 2022 Nov 2;17(11):e0276944. doi: 10.1371/journal.pone.0276944 (PMC9629616; doi:10.1371/journal.pone.0276944)
Supplement: S1 Table — (DOCX) [file pone.0276944.s002.docx]

**Supplementary Material** **Table 1. The GMM Model of PAH and blood routine indexes**

|  | | PLT | PDW | MPV | PCT | PLCR |
| --- | --- | --- | --- | --- | --- | --- |
| variable | | F(P) | F(P) | F(P) | F(P) | F(P) |
| 2-OHNa |  |  |  |  |  |  |
|  | group | 0.28 (0.8367) | 0.41 (0.7461) | 0.14 (0.9336) | 0.30 (0.8265) | 0.62 (0.6052) |
|  | time | 1.38 (0.2517) | 0.26 (0.7699) | 0.23 (0.7962) | 0.71 (0.4945) | 0.45 (0.6394) |
|  | group*time | 1.33 (0.2424) | 1.23 (0.2874) | 0.89 (0.5027) | 0.33 (0.9230) | 0.32 (0.9253) |
|  | 2-OHNa*time | 0.56 (0.5709) | 0.35 (0.7026) | 0.72 (0.4891) | 0.07 (0.9293) | 0.08 (0.9262) |
|  | 2-OHNa*group | 0.68 (0.5643) | 1.03 (0.3787) | 1.05 (0.3716) | 0.52 (0.6686) | 0.47 (0.7041) |
|  | 2-OHNa*group*time | 0.71 (0.6393) | 1.13 (0.3447) | 0.88 (0.5099) | 0.71 (0.6441) | 0.79 (0.5797) |
| 1-OHNa |  |  |  |  |  |  |
|  | group | 0.35 (0.7913) | 1.50 (0.2162) | 0.95 (0.4150) | 0.51 (0.6737) | 1.44 (0.2317) |
|  | time | 1.25 (0.2883) | 0.25 (0.7786) | 0.33 (0.7175) | 0.78 (0.4591) | 0.59 (0.5532) |
|  | group*time | 1.37 (0.2241) | 0.51 (0.8037) | 0.28 (0.9483) | 0.17 (0.9840) | 0.38 (0.8894) |
|  | 1-OHNa*time | 0.07 (0.9291) | 0.05 (0.9543) | 0.12 (0.8911) | 1.24 (0.2915) | 0.28 (0.7579) |
|  | 1-OHNa*group | 0.85 (0.4663) | 0.14 (0.9376) | 0.25 (0.8634) | 0.34 (0.7937) | 0.23 (0.8784) |
|  | 1-OHNa*group*time | 3.10 (0.0056)* | 1.01 (0.4166) | 0.95 (0.4604) | 0.48 (0.8237) | 0.61 (0.7222) |
| 9-OHFlu |  |  |  |  |  |  |
|  | group | 0.91 (0.4367) | 0.31 (0.8195) | 0.32 (0.8122) | 0.38 (0.7667) | 0.17 (0.9156) |
|  | time | 0.87 (0.4194) | 0.63 (0.5327) | 0.66 (0.5179) | 0.66 (0.5153) | 0.71 (0.4941) |
|  | group*time | 1.30 (0.2566) | 3.51 (0.0021)* | 3.68 (0.0014)* | 0.91 (0.4900) | 1.09 (0.3659) |
|  | 9-OHFlu*time | 0.01 (0.9948) | 0.14 (0.8664) | 0.16 (0.8536) | 0.07 (0.9369) | 0.22 (0.8044) |
|  | 9-OHFlu*group | 0.95 (0.4158) | 0.87 (0.4559) | 0.54 (0.6530) | 0.67 (0.5708) | 0.61 (0.6066) |
|  | 9-OHFlu*group*time | 1.03 (0.4058) | 3.09 (0.0057)* | 3.42 (0.0026)* | 1.60 (0.1461) | 1.02 (0.4133) |
| 2-OHFlu |  |  |  |  |  |  |
|  | group | 0.08 (0.9713) | 1.12 (0.3433) | 0.71 (0.5493) | 0.47 (0.7060) | 1.22 (0.3025) |
|  | time | 1.59 (0.2054) | 0.21 (0.8143) | 0.29 (0.7515) | 0.82 (0.4424) | 0.40 (0.6685) |
|  | group*time | 1.19 (0.3129) | 1.28 (0.2662) | 1.02 (0.4130) | 0.30 (0.9383) | 0.30 (0.9379) |
|  | 2-OHFlu*time | 0.21 (0.8084) | 0.21 (0.8114) | 0.41 (0.6623) | 0.04 (0.9607) | 0.04 (0.9578) |
|  | 2-OHFlu*group | 0.07 (0.9768) | 0.48 (0.6954) | 0.18 (0.9097) | 0.75 (0.5255) | 0.32 (0.8101) |
|  | 2-OHFlu*group*time | 0.66 (0.6783) | 0.69 (0.6578) | 0.50 (0.8098) | 0.60 (0.7311) | 0.43 (0.8609) |
| 2-OHPh |  |  |  |  |  |  |
|  | group | 0.02 (0.9950) | 1.75 (0.1576) | 1.03 (0.3782) | 0.22 (0.8790) | 2.04 (0.1094) |
|  | time | 1.59 (0.2058) | 0.58 (0.5578) | 0.71 (0.4937) | 1.03 (0.3587) | 0.59 (05552) |
|  | group*time | 0.67 (0.6721) | 1.12 (0.3475) | 0.82 (0.5526) | 0.09 (0.9977) | 0.33 (0.9189) |
|  | 2-OHPh*time | 0.20 (0.8186) | 0.18 (0.8328) | 0.28 (0.7597) | 0.06 (0.9447) | 0.09 (0.9123) |
|  | 2-OHPh*group | 0.28 (0.8388) | 0.29 (0.8325) | 0.30 (0.8225) | 0.62 (0.6011) | 0.32 (0.8074) |
|  | 2-OHPh*group*time | 0.70 (0.6487) | 0.07 (0.9988) | 0.08 (0.9982) | 0.63 (0.7089) | 0.30 (0.9359) |
| 1-OHPh |  |  |  |  |  |  |
|  | group | 0.11 (0.9566) | 1.80 (0.1473) | 1.16 (0.3273) | 0.12 (0.9495) | 1.65 (0.1795) |
|  | time | 1.20 (0.3035) | 0.84 (0.4324) | 1.10 (0.3336) | 1.15 (0.3162) | 0.46 (0.6290) |
|  | group*time | 0.57 (0.7574) | 1.25 (0.2819) | 0.95 (0.4564) | 0.02 (1.0000) | 0.30 (0.9370) |
|  | 1-OHPh*time | 0.81 (0.4464) | 0.41 (0.6640) | 0.65 (0.5243) | 0.26 (0.7739) | 0.08 (0.9224) |
|  | 1-OHPh*group | 0.58 (0.6276) | 0.26 (0.8541) | 0.37 (0.7743) | 0.28 (0.8423) | 0.04 (0.9904) |
|  | 1-OHPh*group*time | 0.59 (0.7380) | 0.24 (0.9649) | 0.17 (0.9843) | 0.59 (0.7355) | 0.08 (0.9977) |
| 1-OHP |  |  |  |  |  |  |
|  | group | 0.20 (0.8986) | 1.57 (0.1967) | 1.33 (0.2656) | 0.19 (0.9004) | 1.68 (0.1727) |
|  | time | 0.54 (0.5815) | 0.80 (0.4495) | 1.14 (0.3203) | 0.82 (0.4416) | 0.86 (0.4240) |
|  | group*time | 0.55 (0.7698) | 1.08 (0.3749) | 0.97 (0.4421) | 0.25 (0.9575) | 0.51 (0.8011) |
|  | 1-OHP*time | 0.75 (0.4745) | 0.28 (0.7560) | 0.74 (0.4790) | 0.74 (0.4756) | 0.24 (0.7899) |
|  | 1-OHP*group | 0.49 (0.6916) | 0.84 (0.4740) | 0.81 (0.4875) | 0.20 (0.8939) | 0.20 (0.8976) |
|  | 1-OHP*group*time | 1.23 (0.2890) | 0.99 (0.4347) | 0.93 (0.4701) | 1.06 (0.3840) | 0.68 (0.6627) |
| 3-OHBaPB |  |  |  |  |  |  |
|  | group | 0.17 (0.9174) | 1.51 (0.2123) | 0.68 (0.5625) | 0.06 (0.9811) | 0.18 (0.9111) |
|  | time | 1.62 (0.2000) | 0.99 (0.3730) | 1.13 (0.3228) | 0.66 (0.5166) | 0.42 (0.6585) |
|  | group*time | 1.02 (0.4127) | 0.44 (0.8548) | 0.35 (0.9081) | 0.31 (0.9322) | 0.77 (0.5944) |
|  | 3-OHBaPB*time | 0.33 (0.7194) | 0.59 (0.5543) | 0.79 (0.4530) | 0.05 (0.9495) | 0.55 (0.5799) |
|  | 3-OHBaPB*group | 0.44 (0.7211) | 0.12 (0.9495) | 0.02 (0.9972) | 0.30 (0.8272) | 1.34 (0.2601) |
|  | 3-OHBaPB*group*time | 1.05 (0.3892) | 0.45 (0.8482) | 0.66 (0.6806) | 0.56 (0.7597) | 2.00 (0.0639) |
