# Supplementary material for: Long-term exposure to low concentrations of polycyclic aromatic hydrocarbons and alterations in platelet indices: A longitudinal study in China
Source: PLoS One. 2022 Nov 2;17(11):e0276944. doi: 10.1371/journal.pone.0276944 (PMC9629616; doi:10.1371/journal.pone.0276944)
Supplement: S2 Table — Notes, the confidence and 95% confidence interval result of the models have listed in this each cell of the table, by the order from Model 1 to Model 3. (DOCX) [file pone.0276944.s003.docx]

**Supplementary Material Table 2 The GLMM Model of PAH and PLT**

| Variable | Level | 1-OHNa | 2-OHFlu | 2-OHPh | 1-OHPh | 1-OHP |
| --- | --- | --- | --- | --- | --- | --- |
| Group  (Level 4 is reference) | 1 | -0.0111(-0.0577,0.0354)/  -0.0139(-0.0616,0.0338)/  -0.0113(-0.0594,0.0369) | -0.0089(-0.0558,0.038)/  -0.0112(-0.0593,0.0369)/  -0.0091(-0.0576,0.0395) | -0.0098(-0.0566,0.037)/  -0.0116(-0.0596,0.0363)/  -0.0093(-0.0577,0.0392) | -0.011(-0.058,0.0359)/  -0.0129(-0.061,0.0353)/  -0.0104(-0.059,0.0381) | -0.008(-0.0547,0.0387)/  -0.0113(-0.0591,0.0365)/  -0.0092(-0.0575,0.039) |
|  | 2 | 0.0049(-0.04,0.0499)/  0.0032(-0.0427,0.0492)/  0.0046(-0.0415,0.0508) | 0.0048(-0.0405,0.0501)/  0.0036(-0.0427,0.0499)/  0.0048(-0.0418,0.0514) | 0.0065(-0.0387,0.0517)/  0.0057(-0.0405,0.0519)/  0.0071(-0.0394,0.0536) | 0.0032(-0.0421,0.0485)/  0.0025(-0.0438,0.0488)/  0.0039(-0.0427,0.0505) | 0.004(-0.041,0.049)/  0.002(-0.0439,0.048)/  0.003(-0.0432,0.0492) |
|  | 3 | -0.0038(-0.0514,0.0437)/  -0.0047(-0.0529,0.0435)/  -0.0052(-0.0535,0.0432) | -0.0011(-0.0489,0.0468)/  -0.0013(-0.0498,0.0472)/  -0.0016(-0.0504,0.0471) | -0.002(-0.0498,0.0457)/  -0.0019(-0.0503,0.0465)/  -0.0023(-0.0509,0.0463) | -0.0027(-0.0506,0.0453)/  -0.0029(-0.0516,0.0458)/  -0.0033(-0.0522,0.0455) | -0.0008(-0.0483,0.0467)/  -0.002(-0.0501,0.0462)/  -0.0023(-0.0506,0.046) |
| Time  (Level 3 is reference) | 1 | -0.025(-0.0578,0.0077)/  -0.0248(-0.0577,0.0082)/  -0.0248(-0.0577,0.0082) | -0.0252(-0.0577,0.0073)/  -0.025(-0.0577,0.0076)/  -0.0251(-0.0578,0.0076) | -0.024(-0.0559,0.0078)/  -0.0238(-0.0559,0.0082)/  -0.0239(-0.056,0.0082) | -0.0258(-0.0581,0.0065)/  -0.0253(-0.0578,0.0072)/  -0.0253(-0.0578,0.0072) | -0.0249(-0.0576,0.0078)/  -0.0245(-0.0574,0.0084)/  -0.0245(-0.0574,0.0084) |
|  | 2 | -0.0295(-0.0637,0.0047)/  -0.0294(-0.0637,0.0048)/  -0.0294(-0.0636,0.0049) | -0.0294(-0.0637,0.005)/  -0.0293(-0.0638,0.0051)/  -0.0294(-0.0639,0.0051) | -0.0281(-0.0627,0.0064)/  -0.0281(-0.0627,0.0065)/  -0.0281(-0.0628,0.0065) | -0.0299(-0.0643,0.0045)/  -0.0297(-0.0642,0.0048)/  -0.0297(-0.0642,0.0048) | -0.0297(-0.0641,0.0047)/  -0.0296(-0.0641,0.005)/  -0.0296(-0.0641,0.0049) |
| age |  | -/0.0021(-0.0317,0.0359)/  0.0015(-0.0325,0.0355) | -/0.0013(-0.0326,0.0352)/  0.0009(-0.0333,0.0351) | -/0.002(-0.0318,0.0358)/  0.0017(-0.0323,0.0358) | -/0.0042(-0.0298,0.0382)/  0.0038(-0.0305,0.0381) | -/0.003(-0.0309,0.0369)/  0.0022(-0.0319,0.0364) |
| sex |  | -/-0.008(-0.0421,0.026)/  -0.0035(-0.0384,0.0315) | -/-0.0108(-0.0454,0.0238)/  -0.0071(-0.0426,0.0285) | -/0.002(-0.0318,0.0358)/  0.0017(-0.0323,0.0358) | -/-0.0105(-0.0448,0.0239)/  -0.0062(-0.0414,0.029) | -/-0.0094(-0.0433,0.0245)/  -0.0057(-0.0405,0.0291) |
| BMI |  | -/-0.0085(-0.0486,0.0316)  /-0.0075(-0.0478,0.0328) | -/-0.0053(-0.0458,0.0353)/  -0.0047(-0.0455,0.0361) | -/-0.0034(-0.0437,0.037)/  -0.0028(-0.0434,0.0378) | -/-0.0034(-0.0437,0.037)/  -0.0028(-0.0434,0.0378) | -/-0.0115(-0.0516,0.0286)/  -0.0104(-0.0507,0.03) |
| Smoking |  | -/-/0.0033(-0.0315,0.038) | -/-/0.0019(-0.0329,0.0367) | -/-/0.0072(-0.0275,0.0418) | -/-/0.0011(-0.0337,0.036) | -/-/0.0042(-0.0304,0.0388) |
| Drinking |  | -/-/-0.0077(-0.0494,0.033) | -/-/-0.0031(-0.0453,0.039) | -/-/-0.0028(-0.0449,0.0393) | -/-/-0.0031(-0.0452,0.0389) | -/-/-0.0084(-0.0499,0.0332) |
| Matesmoke |  | -/-/0.0113(-0.0039,0.0265) | -/-/0.0091(-0.0063,0.0244) | -/-/0.0099(-0.0054,0.0252) | -/-/0.0103(-0.005,0.0257) | -/-/0.0099(-0.0053,0.0251) |

Notes, the confidence and 95% confidence interval result of the models have listed in this each cell of the table, by the order from Model 1 to Model 3.
