# Supplementary material for: Long-term exposure to low concentrations of polycyclic aromatic hydrocarbons and alterations in platelet indices: A longitudinal study in China
Source: PLoS One. 2022 Nov 2;17(11):e0276944. doi: 10.1371/journal.pone.0276944 (PMC9629616; doi:10.1371/journal.pone.0276944)
Supplement: S3 Table — Notes, the confidence and 95% confidence interval result of the models have been listed in each cell of the table, by the order from Model 1 to Model 3. (DOCX) [file pone.0276944.s004.docx]

**Supplementary Material Table 3. The GLMM Model of 9-OHFlu and three platelet indices**

| Variable | Level | PDW | MPV | P-LCR |
| --- | --- | --- | --- | --- |
| Group  (Level 4 is reference) | 1 | 0.0375(-0.0044,0.0793)/0.0355(-0.0073,0.0782)/  0.0393(-0.0035,0.0821) | 0.0323(-0.0227,0.0873)/0.0324(-0.0239,0.0886)/  0.0376(-0.0188,0.0939) | 0.0128(-0.001,0.0266)/0.0115(-0.0026,0.0256)/  0.0135(-0.0003,0.0274) |
|  | 2 | 0.04064*(0.0003,0.081)/0.0387(-0.0024,0.0799)/  0.0407(-0.0004,0.0817) | 0.0405(-0.0126,0.0935)/0.0404(-0.0137,0.0945)/  0.043(-0.011,0.0969) | 0.0126(-0.0007,0.0259)/0.0121(-0.0014,0.0257)/  0.0129(-0.0005,0.0262) |
|  | 3 | 0.0162(-0.0266,0.0591)/0.0147(-0.0286,0.0579)/  0.014(-0.0291,0.0571) | 0.0192(-0.037,0.0755)/0.0198(-0.0372,0.0768)/  0.0187(-0.038,0.0754) | 0.0023(-0.0119,0.0164)/0.0018(-0.0124,0.016)/  0.0016(-0.0123,0.0155) |
| Time  (Level 3 is reference) | 1 | 0.0059(-0.0179,0.0298)/0.0059(-0.0182,0.0299)/  0.0057(-0.0184,0.0298) | 0.0095(-0.0179,0.037)/0.0093(-0.0185,0.037)/  0.0091(-0.0187,0.0369) | 0.0055(-0.005,0.016)/0.006(-0.0046,0.0165)/  0.0059(-0.0047,0.0164) |
|  | 2 | -0.0064(-0.0344,0.0216)/-0.0064(-0.0346,0.0217)/  -0.0065(-0.0346,0.0216) | -0.0078(-0.0411,0.0256)/-0.0079(-0.0413,0.0255)/  -0.008(-0.0414,0.0255) | 0.0024(-0.0084,0.0132)/0.0026(-0.0083,0.0134)/  0.0025(-0.0084,0.0134) |
| age |  | -/-0.0014(-0.031,0.0282)/-0.00287(-0.0326,0.0268) | -/-0.0029(-0.0408,0.035)/-0.0044(-0.0424,0.0336) | -/0.0043(-0.006,0.0146)/0.003(-0.0072,0.0132) |
| sex |  | -/0.0048(-0.0256,0.0352)/0.0105(-0.0204,0.0414) | -/-0.0033(-0.0433,0.0366)/0.005(-0.0357,0.0457) | -/-0.0045(-0.0144,0.0055)/-0.0018(-0.0118,0.0082) |
| BMI |  | -/-0.0094(-0.0433,0.0244)/-0.008(-0.0419,0.0258) | -/0.0015(-0.0406,0.0436)/0.0028(-0.0393,0.045) | -/-0.004(-0.016,0.008)/-0.0037(-0.0156,0.0082) |
| Smoking |  | -/-/0.0088(-0.0219,0.0396) | -/-/0.0089(-0.0316,0.0494) | -/-/0.0062(-0.0037,0.0161) |
| Drinking |  | -/-/-0.0113(-0.0484,0.0258) | -/-/-0.0126(-0.0614,0.0363) | -/-/-0.0027(-0.0147,0.0093) |
| Matesmoke |  | -/-/0.0142(0.0011,0.0273) | -/-/0.0203(0.0031,0.0374) | -/-/0.0076(0.0034,0.0118) |
| 9-OHFlu  (Level 4 is reference) | 1 | -0.043(-0.073 -0.012)/-0.043(-0.074 -0.012)/  -0.042(-0.073 -0.012) | -0.057(-0.094 -0.021)/-0.057(-0.094 -0.021)/  -0.056(-0.093 -0.02) | -0.014(-0.026 -0.003)/-0.015(-0.026 -0.003)/  -0.015(-0.026 -0.004) |
|  | 2 | -0.011(-0.045 0.023)/-0.011(-0.045 0.023)/  -0.012(-0.045 0.022) | -0.008(-0.048 0.033)/-0.007(-0.048 0.033)/  -0.007(-0.048 0.033) | -0.003(-0.016 0.009)/-0.004(-0.017 0.009)/  -0.005(-0.017 0.008) |
|  | 3 | 0.027(-0.005 0.059)/0.027(-0.006 0.059)/  0.027(-0.005 0.059) | 0.025(-0.014 0.063)/0.025(-0.013 0.064)/  0.026(-0.013 0.064) | 0.011(-0.001 0.023)/0.011(-0.001 0.023)/  0.011(-0.001 0.023) |

Notes, the confidence and 95% confidence interval result of the models have been listed in each cell of the table, by the order from Model 1 to Model 3.
