# Supplementary material for: Long-term exposure to low concentrations of polycyclic aromatic hydrocarbons and alterations in platelet indices: A longitudinal study in China
Source: PLoS One. 2022 Nov 2;17(11):e0276944. doi: 10.1371/journal.pone.0276944 (PMC9629616; doi:10.1371/journal.pone.0276944)
Supplement: S4 Table — Note, we collected the variables from each of the 222 patients in these three aspects. The variables in the latter two aspects have been collected three times for repeated measures. While list their full names, their acronyms have been listed in the brackets for the convenience of further use. (DOCX) [file pone.0276944.s005.docx]

**Supplementary Material Table 4. The variables collected from all 222 participants**

| **Name of the variables** |
| --- |
| **Demographic Information** |
| Sex(male/female), Age, Height, Weight, Whether work in , Smoking, Alcohol consumption, Drinking frequency, Whether his/her mate smoke(MS), Barbecue, Barbecue frequency, Body Mass Index(BMI) |
| **Blood Routine Indexes(BRI)** |
| **Thrombocyte indexes:**  Count of platelet(PLT), Platelet distribution width(PDW), mean platelet volume(MPV), platelet crit(PCT),  large platelet ratio(P-LCR) |
| **Metabolites of Polycyclic Aromatic Hydrocarbons in Urine (MPAHU)** |
| 2-hydroxynaphthalene(2-OHNa), 1-hydroxynaphthalene(1-OHNa), 2-hydroxyfluorene (2-OHFlu),  9-hydroxyfluorene (9-OHFlu), 2-hydroxyphenanthrene (2-OHPh), 1-hydroxyphenanthrene (1-OHPh),  1-hydroxypyrene (1-OHP), 3-hydroxybenzo[a]pyrene(3-OHBaP) |

Note, we collected the variables from each of the 222 patients in these three aspects. The variables in the latter two aspects have been collected three times for [repeated](javascript:;) [measure](javascript:;)s. While list their full names, their [acronym](javascript:;)s have been listed in the brackets for the convenience of further use.
