# Supplementary material for: Long-term exposure to low concentrations of polycyclic aromatic hydrocarbons and alterations in platelet indices: A longitudinal study in China
Source: PLoS One. 2022 Nov 2;17(11):e0276944. doi: 10.1371/journal.pone.0276944 (PMC9629616; doi:10.1371/journal.pone.0276944)
Supplement: S5 Table — Note, PLT is short for the count of platelet, while PDW for Platelet distribution width, MPV for mean platelet volume, PCT for platelet crit, P-LCR for large platelet ratio, 2-OHNa for 2-hydroxynaphthalene, 1-OHNa for 1-hydroxynaphthalene, 2-OHFlu for 2-hydroxyfluorene, 9-OHFlu for 9-hydroxyfluorene, 2-OHPh for 2-hydroxyphenanthrene, 1-OHPh for 1-hydroxyphenanthrene, 1-OHP for 1-hydroxypyrene, 3-OHBaP for 3-hydroxybenzo[a]pyrene. Listing the geometric mean± geometric standard error for each following-up time of each index, the p values according to Mauchly’s test of sphericity have been recorded in the fourth row of each group with "ST" for short. The last row of each group listed the results of ANOVA for repeated measurement (MANOVA) or traditional ANOVA analysis while their P values have been displayed in the brackets after F values. * stands for the statistical significance with α = 0.05. (DOCX) [file pone.0276944.s006.docx]

**Supplementary Material Table 5.** **The variates of platelet indices and UPAHM among times**

| Group | Var | PLT | PDW | MPV | PCT | PLCR | 2-OHNa | 1-OHNa | 9-OHFlu | 2-OHFlu | 2-OHPh | 1-OHPh | 1-OHP | 3-OHBaP |
| --- | --- | --- | --- | --- | --- | --- | --- | --- | --- | --- | --- | --- | --- | --- |
| First | 1 | 223.24±87.00 | 11.21±3.34 | 9.53±2.35 | 0.23±0.08 | 23.68±9.05 | 0.62±0.86 | 0.92±0.93 | 0.97±0.85 | 0.28±0.64 | -0.13±0.70 | -0.18±0.65 | -0.37±0.67 | -0.53±0.73 |
|  | 2 | 203.98±88.71 | 10.48±3.83 | 8.99±3.07 | 0.21±0.09 | 24.84±24.71 | 0.39±0.81 | 0.77±0.90 | 0.89±0.79 | 0.10±0.65 | -0.24±0.68 | -0.25±0.68 | -0.51±0.76 | -0.61±0.75 |
|  | 3 | 241.14±88.54 | 11.02±3.42 | 9.38±2.61 | 0.23±0.08 | 23.14±9.19 | 0.31±0.74 | 0.94±0.90 | 1.06±0.81 | 0.20±0.75 | -0.20±0.75 | -0.30±0.72 | -0.47±0.91 | -0.50±0.71 |
|  | ST | 0.6383 | 0.0141 | 0.0001 | 0.2596 | <0.0001 | 0.115 | 0.5949 | 0.8968 | 0.0164 | 0.0435 | 0.1651 | 0.0215 | 0.9483 |
|  | F (p) | 2.58(0.08) | 0.82(0.42) | 0.84(0.40) | 0.55(0.57) | 0.19(0.73) | 2.27(0.11) | 0.84(0.43) | 0.57(0.56) | 1.20(0.31) | 0.47(0.61) | 0.53(0.59) | 0.62(0.52) | 0.37(0.69) |
| Second | 1 | 215.84±84.97 | 10.54±3.15 | 9.22±2.46 | 0.23±0.08 | 24.38±23.30 | 0.53±0.77 | 0.65±0.86 | 0.88±0.83 | 0.16±0.60 | -0.20±0.68 | -0.19±0.60 | -0.38±0.62 | -0.59±0.71 |
|  | 2 | 234.88±73.54 | 11.32±3.17 | 9.57±2.27 | 0.23±0.07 | 23.62±9.02 | 0.27±0.71 | 0.74±0.88 | 0.97±0.81 | 0.10±0.74 | -0.20±0.71 | -0.31±0.69 | -0.49±0.84 | -0.42±0.61 |
|  | 3 | 229.67±77.51 | 11.32±2.61 | 9.67±1.89 | 0.23±0.07 | 24.03±7.84 | 0.57±0.71 | 0.91±0.95 | 0.78±0.71 | 0.29±0.62 | 0.05±0.55 | -0.05±0.54 | -0.23±0.45 | -0.51±0.73 |
|  | ST | 0.264 | 0.2286 | 0.1744 | 0.3933 | <0.0001 | 0.3599 | 0.9649 | 0.1981 | 0.282 | 0.0976 | 0.6931 | 0.0404 | 0.8247 |
|  | F (p) | 0.85(0.43) | 1.35(0.27) | 0.73(0.49) | 0.20(0.82) | 0.04(0.89) | 4.85(0.01)* | 1.46(0.24) | 1.25(0.29) | 1.53(0.22) | 3.50(0.04)* | 3.84(0.03)* | 3.12(0.05) | 1.06(0.35) |
| Third | 1 | 221.96±70.17 | 11.09±2.00 | 9.64±1.54 | 0.23±0.06 | 23.23±6.11 | 0.30±0.79 | 0.86±0.94 | 0.98±0.81 | 0.10±0.69 | -0.16±0.69 | -0.36±0.68 | -0.48±0.67 | -0.55±0.67 |
|  | 2 | 225.27±88.76 | 10.70±3.03 | 9.31±2.47 | 0.21±0.08 | 22.65±8.07 | 0.60±0.66 | 1.13±0.92 | 0.91±0.75 | 0.39±0.59 | 0.08±0.60 | -0.05±0.60 | -0.28±0.52 | -0.42±0.58 |
|  | 3 | 232.65±96.28 | 10.27±3.12 | 8.97±2.73 | 0.22±0.09 | 20.96±7.87 | 0.66±0.66 | 1.02±1.09 | 0.84±0.76 | 0.42±0.58 | 0.09±0.63 | 0.02±0.61 | -0.16±0.45 | -0.52±0.70 |
|  | ST | 0.3735 | 0.0078 | 0.003 | 0.1172 | 0.5347 | 0.7573 | 0.5221 | 0.6228 | 0.4274 | 0.4299 | 0.1559 | 0.1206 | 0.5153 |
|  | F (p) | 0.24(0.79) | 1.31(0.27) | 1.51(0.23) | 0.67(0.51) | 1.50(0.23) | 4.27(0.02)* | 1.34(0.27) | 0.52(0.60) | 4.61(0.01)* | 2.97(0.06) | 5.33(0.01)* | 3.83(0.03)* | 0.68(0.51) |
| Fourth | 1 | 231.85±85.87 | 10.59±3.51 | 9.14±2.82 | 0.22±0.08 | 22.16±9.16 | 0.57±0.86 | 0.92±0.90 | 0.68±0.74 | 0.34±0.64 | -0.07±0.70 | -0.12±0.59 | -0.33±0.55 | -0.59±0.77 |
|  | 2 | 212.74±100.36 | 9.73±3.72 | 8.68±3.09 | 0.21±0.10 | 19.81±9.16 | 0.53±0.63 | 0.94±1.04 | 0.81±0.79 | 0.30±0.50 | -0.03±0.56 | -0.19±0.61 | -0.27±0.43 | -0.66±0.78 |
|  | 3 | 236.33±102.31 | 10.06±3.73 | 8.83±3.11 | 0.22±0.09 | 20.85±9.01 | 0.40±0.76 | 0.90±1.00 | 0.82±0.75 | 0.27±0.69 | -0.00±0.77 | -0.12±0.63 | -0.28±0.54 | -0.72±0.81 |
|  | ST | 0.7258 | 0.4317 | 0.3941 | 0.2224 | 0.1377 | 0.3417 | 0.3222 | 0.8852 | 0.2258 | 0.192 | 0.2993 | 0.3576 | 0.3535 |
|  | F (p) | 0.92(0.41) | 1.19(0.32) | 0.57(0.57) | 0.29(0.74) | 1.48(0.24) | 0.64(0.53) | 0.02(0.98) | 0.46(0.63) | 0.09(0.91) | 0.10(0.90) | 0.22(0.80) | 0.20(0.82) | 0.21(0.81) |

Note, PLT is short for the count of platelet, while PDW for Platelet distribution width, MPV for mean platelet volume, PCT for platelet crit, P-LCR for large platelet ratio, 2-OHNa for 2-hydroxynaphthalene, 1-OHNa for 1-hydroxynaphthalene, 2-OHFlu for 2-hydroxyfluorene, 9-OHFlu for 9-hydroxyfluorene, 2-OHPh for 2-hydroxyphenanthrene, 1-OHPh for 1-hydroxyphenanthrene, 1-OHP for 1-hydroxypyrene, 3-OHBaP for 3-hydroxybenzo[a]pyrene. Listing the [geometric](javascript:;) [mean](javascript:;)± [geometric](javascript:;) standard error for each following-up time of each index, the p values according to Mauchly's test of sphericity have been recorded in the fourth row of each group with "ST" for short. The last row of each group listed the results of ANOVA for repeated measurement (MANOVA) or traditional ANOVA analysis while their P values have been displayed in the [bracket](javascript:;)s after F values. * stands for the statistical significance with α=0.05.
