# Supplementary material for: Long-term exposure to low concentrations of polycyclic aromatic hydrocarbons and alterations in platelet indices: A longitudinal study in China
Source: PLoS One. 2022 Nov 2;17(11):e0276944. doi: 10.1371/journal.pone.0276944 (PMC9629616; doi:10.1371/journal.pone.0276944)
Supplement: S1 File — (DOCX) [file pone.0276944.s008.docx]

**1.The introduction of GLMM and GMM**

Generalized Linear Mixed Model (GLMM)is considered to have unique advantages in dealing with repeated measurement data because it borrows the idea of mixed model. GLMM is not only good at dealing with repeated measurements, it can also be used for data in any hierarchy. GLMM is an extended form of generalized linear model, so that both fixed and random effects can be included[1].

Let $y_{i}$ denote the response variable of the $ith$ individual is made up of subrepeated measurements of the vector. And $x_{i}$ denote the $n_{i}\times p$ covariant matrix for the $ith$ individual，as well as $z_{i}$ denote the $n_{i}\times p$ covariant matrix composed of some covariables, which are usually the covariables with obvious individual variation in $X_{i}$，where $\text{i}=\text{1，2，}\cdots\text{，n}.$
$$f\left( y_{i} \right)=f\left( y_{i}\left| \omega_{i} \right. \right)=exp\left\{ y_{i}\omega_{i}-b\left( \omega_{i} \right)+e\left( y_{i} \right) \right\}$$

Where $\omega_{i}$ is the canonical parameter,$b\left( \omega_{i} \right)$and $e\left( y_{i} \right)$ are known parameters determined by the distribution.Under the GLMM framework, the above statistics have the following relations:

$$\omega_{i}=x_{i}\beta+z_{i}\alpha_{i},\mu\left( \omega_{i} \right)=\mu\left( x_{i}\beta+z_{i}\alpha_{i} \right)$$

Gaussian mixture Model (GMM) is a linear combination of the multiple Gaussian distribution function, theoretical GMM can fit any type of distribution, is often used to solve the same set of data contains a number of different distribution of status or the same class distribution but different parameters, or different types of distribution, such as normal distribution, and Bernoulli distribution[2].

The core idea of the Gaussian mixture model (GMM)is to assume that the data can be regarded as generated from multiple Gaussian distributions. Under this assumption, each separate sub-model is a standard Gaussian model, and its mean and variance are parameters to be estimated.

Let $x_{j}$ denote the $jth$ observation data,$j=1,2,\ldots,N$;K is the number of sub-Gaussian models in a mixed model，$k=1,2,\ldots,K$;$\alpha_{k}$ is the probability that the observed data belongs to the $kth$ sub-model,$\alpha_{k}\geq0,\sum_{k=1}^{K} \alpha_{k}=1$;$\emptyset\left( x\left| \theta_{k} \right. \right)$ is the Gaussian distribution density function of the $kth$sub-model ,$\theta_{k}=\left( \mu_{k},\sigma_{k}^{2} \right)$;$\gamma_{jk}$ denote the probability that the $jth$ observation data belongs to the $kth$ sub-model.

The probability distribution of the Gaussian mixture model is:

$$P\left(\chi\left|\theta\right.\right)=\sum_{\text{k}=1}^{K} \alpha_{\text{k}}\varphi\left(\chi\left|\theta_{\text{k}}\right.\right)$$

For this model, parameters $\theta=\left( \tilde{\mu_{k}},\tilde{\sigma_{k}},\tilde{\alpha_{k}} \right)$ is the expectation of each sub-model, the variance (or covariance), and the probability of occurrence in the mixed model.

**Reference:**

1. Tuerlinckx, F., et al., *Statistical inference in generalized linear mixed models: a review.* Br J Math Stat Psychol, 2006. **59**(Pt 2): p. 225-55.

2. *<GMM_Tutorial_Reynolds.pdf>.*

3. Hughes, D.M., et al., *Dynamic longitudinal discriminant analysis using multiple longitudinal markers of different types.* Stat Methods Med Res, 2018. **27**(7): p. 2060-2080.
